# Ultrathin Nafion-filled porous membrane for zinc/bromine redox flow batteries

**DOI:** 10.1038/s41598-017-10850-9

**Published:** 2017-09-05

**Authors:** Riyul Kim, Hyun Gyu Kim, Gisu Doo, Chanyong Choi, Soohyun Kim, Ju-Hyuk Lee, Jiyun Heo, Ho-Young Jung, Hee-Tak Kim

**Affiliations:** 1 Department of Chemical and Biomolecular Engineering, Korea Advanced Institute of Science and Technology (KAIST), 291, Daehak-ro, Yuseong-gu Daejeon, 34141 Republic of Korea; 2 Advanced Battery Center, KAIST Institute for the NanoCentury, Korea Advanced Institute of Science and Technology (KAIST), 335 Gwahangno, Yuseong-gu Daejeon, 34141 Republic of Korea; 3 Department of Environment & Energy Engineering, Chonnam National University, 77, Yongbong-ro, Buk-gu Gwangju, 61186 Republic of Korea

## Abstract

In this work, we present a 16 μm-thick Nafion-filled porous membrane for Zn/Br redox flow batteries (ZBBs). By using molecular dynamics simulation and dynamic light scattering analysis, we rationally design Nafion solution for Nafion impregnation into a porous polypropylene (PP) separator. A void-free Nafion/PP membrane is successfully fabricated by using NMP as a solvent for the Nafion solution. The resulting membrane shows a smaller area specific resistance in comparison with 600 μm-thick, commercial SF-600 porous membrane. Due to its dense morphology, Br_2_ diffusivity of the Nafion/PP membrane is two orders of magnitude lower than that of SF-600, resulting in a comparable Br_2_ crossover in spite of 37.5 times smaller membrane thickness. As a result, the ZBB based on the Nafion/PP membrane exhibits a higher energy efficiency, demonstrating that ion exchange membrane can outperform the conventional porous membrane by reducing the membrane thickness with inexpensive porous substrate.

## Introduction

For the last decade, there has been increasing interest in large-scale energy storage system (ESS) to meet ever-changing energy supply/demand^1–4^. ESSs, which provide storage of the electric energy from renewable sources and its on-demand release, should be energy-efficient, safe, reliable, and cost-effective. In this regard, redox flow batteries (RFBs) are considered a promising option for the large-scale ESS. RFBs are characterized by their spatial separation of energy storage and energy conversion function, which enables an independent tailoring of power and energy capabilities. Since the positive and negative active materials dissolved in electrolytes are separately stored in individual tanks, safety concerns from the contact of two active materials can be greatly mitigated. Depending on the employed redox couple, RFB takes many forms. Several RFBs have been considered for ESS applications, including all vanadium^5^, zinc/bromine, and iron/chromium RFB. Among these RFBs, the ZBB is one of the most practical options based on its higher energy density (70 Wh kg^−1^) and lower cost^6–8^. The electrochemical reactions of the ZBB are the cathodic deposition of zinc at negative electrode and the anodic formation of a polybromide phase at positive electrode during charging, and the anodic dissolution of zinc at negative electrode and the cathodic formation of Br^−^ at positive electrode during discharging^9–13^ as described in the following equations.

1 $$2{{\rm{Br}}}^{-}\leftrightarrow {{\rm{Br}}}_{2}+2{{\rm{e}}}^{-}({{\rm{E}}}_{0}=1.07\,{\rm{V}}\,{\rm{vs}}{\rm{.}}\,{\rm{SHE}})\,{\rm{at}}\,{\rm{positive}}\,{\rm{electrode}}$$

2 $${{\rm{Zn}}}^{2+}+2{{\rm{e}}}^{-}\leftrightarrow {\rm{Zn}}({{\rm{E}}}_{0}=-0.76\,{\rm{V}}\,{\rm{vs}}{\rm{.}}\,{\rm{SHE}})\,{\rm{at}}\,{\rm{negative}}\,{\rm{electrode}}$$

In conventional ZBB configuration, a porous membrane placing between the positive and negative compartment of ZBB acts as a barrier for Br_2_ crossover, while allowing the ionic conduction of Zn^2+^ and Br^− ^^14, 15^. Until now, several hundred micron-thick hydrophilic-treated porous polyethylene membranes such as SF-600 (Asahi Kasei) and Daramic^®^ membranes have been practically employed considering the balance between ionic conduction and Br_2_ crossover; in order to prevent Br_2_ crossover through the pores, such thick membranes are used in spite of the consequent increase in membrane resistance. On the other hand, non-porous Nafion membranes, which are used for all vanadium RFBs^5^, can also be used for ZBB due to their high bromine blocking ability as demonstrated by Lai *et al*.^8^. They compared Nafion^®^ 115 (127 μm) and Daramic^®^ membrane for ZBB, and found that Nafion^®^ 115 has a higher coulombic efficiency by 15% but a lower voltage efficiency by 12% due to its larger membrane resistance. As a result, Nafion^®^ 115 did not exhibit notable gain in energy efficiency. In addition to the large membrane resistance, the high cost of Nafion membranes can further prevent their use in ZBB.

Against this backdrop, we present a pore-filled composite membrane based on Nafion ionomer and a porous PP separator for ZBB application. The PP separator, which is widely used in the lithium ion battery technology sector, is compatible with ZBBs because of its chemical inertness and mechanical robustness. The role of the PP separator in membrane cost reduction is two-fold; the PP separator is a cost-effective substrate because it is a plentiful commodity, and it can reduce the amount of Nafion material used. In order to achieve a high Br_2_ blocking performance, Nafion ionomer should be impregnated into the pore without forming any voids. In this regard, the solvent for Nafion solution, which is highly important in achieving high-quality Nafion/PP membrane, was rationally designed by using molecular dynamics simulation and dynamic light scattering analysis. In this work, we demonstrate the efficacy of the Nafion/PP membrane for the use in ZBB in comparison with a commercial microporous membrane, SF-600. Due to its low membrane thickness imparted by the PP separator, the Nafion/PP membrane has smaller membrane resistance, and consequently higher voltage efficiency. Because of the dense morphology of the Nafion/PP membrane, Br_2_ crossover can be effectively suppressed even at a thickness 37.5 times smaller than that of SF-600 membrane. As a result, the Nafion/PP membrane-based ZBB exhibits a higher energy efficiency than the SF-600-based one. It is the first demonstration of superior performance of Nafion-based membrane over conventional porous membranes in ZBB.

## Results and Discussion

**Figure 1:**
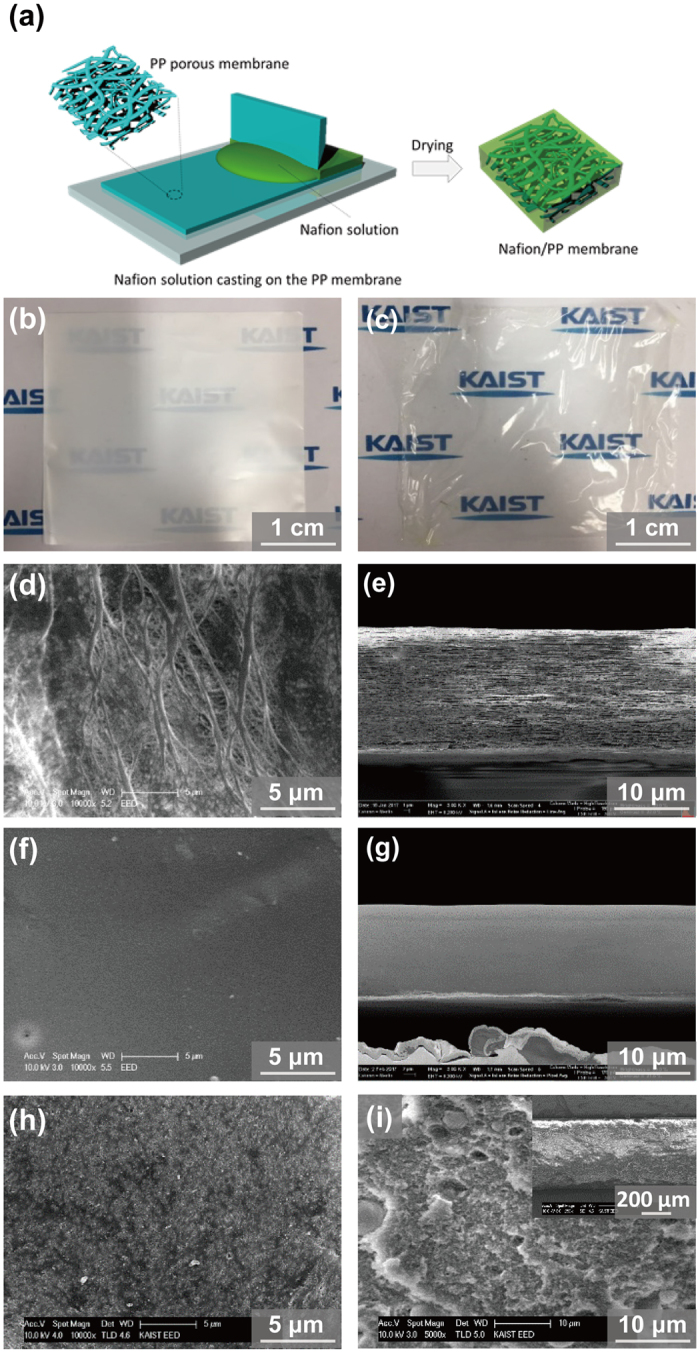
(**a**) Schematic of the Nafion/PP membrane fabrication process. Optical image of (**b**) pristine porous PP membrane and (**c**) Nafion-filled PP membrane. SEM images of pristine porous PP membrane: (**d**) surface and (**e**) cross-section, of the Nafion/PP membrane: (**f**) surface and (**g**) cross-section, and of SF-600 membrane: (**h**) surface and (**i**) cross-section (Inset: a low magnification image).

The fabrication process of the Nafion/PP membrane is illustrated in Fig. 1a. 10 wt% Nafion solution in N-methyl pyrrolidone (NMP) was cast on the PP separator. During the casting, the Nafion solution was impregnated into the pores and, during the subsequent drying process, the Nafion ionomers were solidified with filling the pores. After the impregnation, the opaque PP separator became transparent as shown in Fig. 1b,c, indicating a successful Nafion impregnation. The pristine PP separator with porosity of 60% has the submicron-sized pores among the interconnected polymer fibrils as shown in the surface SEM image (Fig. 1d). The PP fibrils are aligned along the in-plane direction because the PP separator was fabricated by a bi-axial stretching method (Fig. 1e). These pores were densely filled with Nafion ionomer without any voids as demonstrated in Fig. 1f,g. The total thickness of the composite membrane was around 16 μm, including 3 μm-thick Nafion skin layer. The Nafion skin originates from the excess Nafion solution remaining on the PP separator after the impregnation. The resulting membrane was even thinner than the thickness of the PP separator, indicating that the pores in the PP separator were compacted during the drying process. For comparison, the surface and cross-sectional SEM images of 600 μm-thick SF-600 membrane, which is a typical commercial membrane used for ZBB, are also displayed in Fig. 1h,i, respectively. The images feature a highly porous structure with sub-micron pores and SiO_2_ nanoparticles. According to the pore-size distribution of SF-600 (Fig. S1, Supporting Information), the average pore size and porosity are around 28.2 nm and 60% respectively.

The hybrid of Nafion and PP separator is highly effective in reducing the amount of Nafion used. In comparison with 127 μm-thick Nafion 115 membrane, which is widely used in RFB technology sector, the areal weight of Nafion ionomer for the Nafion/PP membrane is about 13 times smaller than that of Nafion 115 because of the large difference in membrane thickness and 60% Nafion volume fraction for the Nafion/PP membrane. Therefore, the cost issue raised by the use of Nafion can be lessened to great extent.

**Figure 2:**
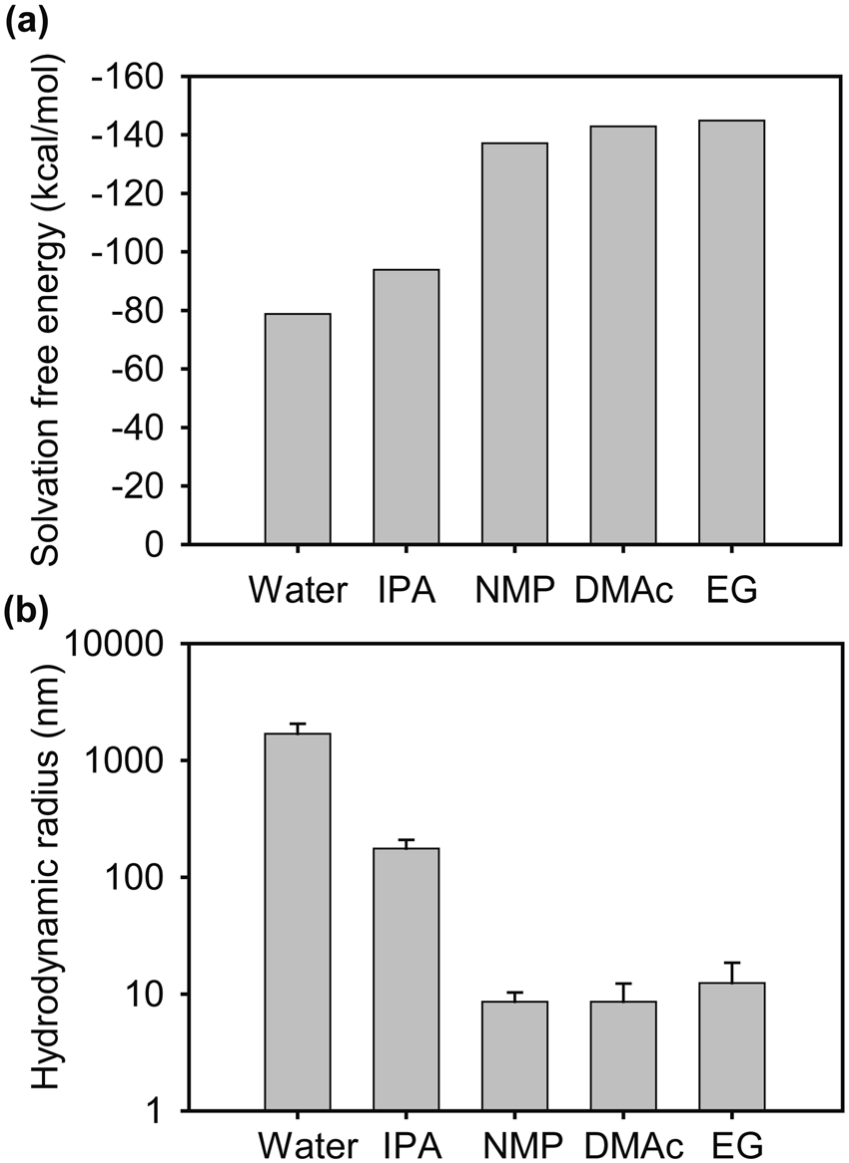
(**a**) Solvation free energy calculation results and (**b**) hydrodynamic radius of Nafion in various casting solvents determined from dynamic light scattering.

For a void-free impregnation of Nafion, the selection of casting solvent presents a challenge. Due to its amphiphilic nature, Nafion tends to form an aggregate in polar solvents such as water and isopropyl alcohol (IPA)^16^. If Nafion aggregates are formed in the casting solution, void-free Nafion filling into the pores is not achievable. Therefore, we tried to find appropriate solvent that can fully dissolve Nafion ionomer based on an atomistic molecular dynamics simulation and dynamic light scattering analysis, which provide a rational design of the casting solvent. Fig. 2a and Fig. S2 compares the solvation free energies of Nafion chain in various solvents including water, IPA, NMP, dimethylacetamide (DMAc), and ethylene glycol (EG). The more negative solvation free energy means the stronger interaction between Nafion chain and solvent. The solvation free energies for NMP, DMAc, and EG were similar but more negative than those for water and IPA. The results indicate that NMP, DMAc and EG are more effective in preventing the formation of Nafion aggregate.

Dynamic light scattering tests for the Nafion solutions with these solvents were conducted to investigate the dimension of Nafion ionomer in these solvents. As shown in Fig. 2b, the hydrodynamic radius of Nafion (R_h_) was smaller than the solvent of more negative solvation free energy. For NMP, DMAc and EG, the values for R_h_ were below 10 nm, which are much smaller than the submicron-sized pores of the PP separator, ensuring that the impregnation of dissolved Nafion chain into the pores. Such small R_h_s indicate a molecular scale dissolution of Nafion chain in the solutions. However, the R_h_s for IPA and water were larger than 100 nm, which are comparable or even larger than the pore size of the PP separator.

As expected from the large R_h_, proper impregnation of Nafion was not obtained with the Nafion solution in IPA even though the PP separator was readily wet with it (Supporting Information, Fig. S3). The Nafion solution in water was also not appropriate for the impregnation due to poor wetting and large Nafion aggregate. These results indicate that the commercial Nafion solutions based on water or water/IPA mixture cannot be directly used for the impregnation. Instead, solidification of Nafion ionomer from the commercial solutions and re-dissolution of the solidified Nafion into the good solvents were necessary to fabricate the Nafion/PP membrane.

In accordance with the simulation and dynamic light scattering results, NMP, DMAc and EG readily dissolved the Nafion ionomer. However, NMP was selected because it exhibited better wettability on the PP separator and proper evaporation rate than DMAc and EG. The casts derived from DMAc and EG were not readily spread on the PP separator, resulting in an incomplete filling of the Nafion into the pores as indicated by the opaque appearance after the impregnation. (Supporting information, Fig. S3). In fact, NMP solvent provided a uniform Nafion filling in the pores due to the nano-scale Nafion dispersion, good wettability on the PP separator, and appropriate evaporation speed.

**Figure 3:**
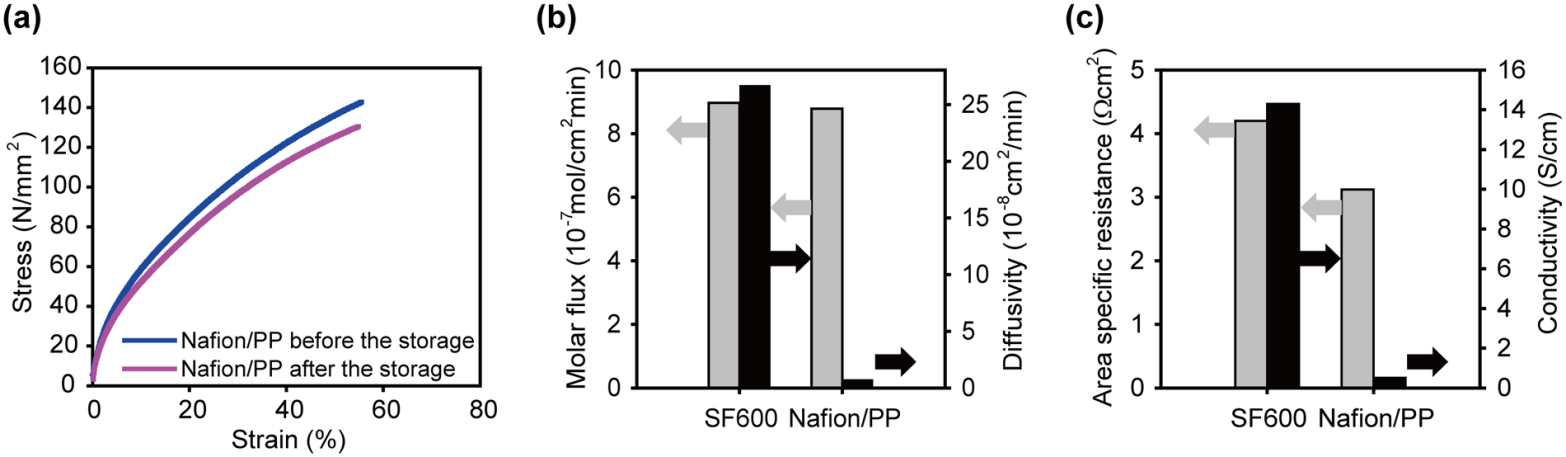
(**a**) Stress-strain curves for the Nafion/PP membrane before and after the Br_2_ storage. (**b**) Br_2_ diffusivity and molar flux of the SF-600 and Nafion/PP membrane. (**c**) Area specific resistance and conductivity of the SF-600 and Nafion/PP membrane equilibrated with a ZBB electrolyte (2.25 M ZnBr_2_ + 0.5 M ZnCl_2_ + 0.8 M MEP + 5 ml L^−1^ Br_2_).

Since ZBB membranes are in contact with highly oxidative Br_2_, the oxidative stability against liquid Br_2_ should be assessed. As shown in Fig. 3a, the stress-strain curves of the Nafion/PP membrane before and after the immersion in Br_2_ solution for 24 h are nearly identical, indicating high chemical stability against liquid Br_2_. The high tensile strength of the Nafion/PP membrane (140 MPa) is contrasted by that of the SF-600 membrane (7.4 MPa) (see Supporting Information Fig. S4). It demonstrates the other benefit of using the mechanically robust PP separator. Since the Zn dendrite grown from the negative electrode often penetrates across the membrane and causes a short-circuit, the mechanical strength imposed by the PP separator can be beneficial in preventing membrane breakdown and achieving stable operation of the ZBB.

The Br_2_ transport properties of the Nafion/PP membrane were investigated and compared with those of SF-600 membrane (Fig. 3b). The values for the Br_2_ molar flux was 8.78 and 8.97 × 10^−7^ mol cm^−2^min^−1^ for Nafion/PP and SF-600 membrane, respectively. Considering the difference in membrane thickness (16 μm for Nafion/PP and 600 μm for SF-600), the Nafion/PP has a two orders of magnitude lower Br_2_ diffusivity (7.53 × 10^−9^ cm^2^min^−1^) than the SF-600 (2.67 × 10^−7^ cm^2^min^−1^), which clearly demonstrates the high Br_2_ blocking ability of the Nafion ionomer. To our interest, the ASR of the Nafion/PP membrane (3.12 Ωcm^2^) is smaller by 25.7% than that of the SF-600 membrane (4.2 Ωcm^2^). Although the Nafion/PP membrane has significantly lower ionic conductivity (Fig. 3c) due to the large difference in liquid electrolyte uptake (14.5% for the Nafion/PP and 168.4% for SF-600), the resistance reduction by the 37.5 times smaller thickness is greater than the resistance increase by the low ionic conductivity, resulting in the smaller ASR.

**Figure 4:**
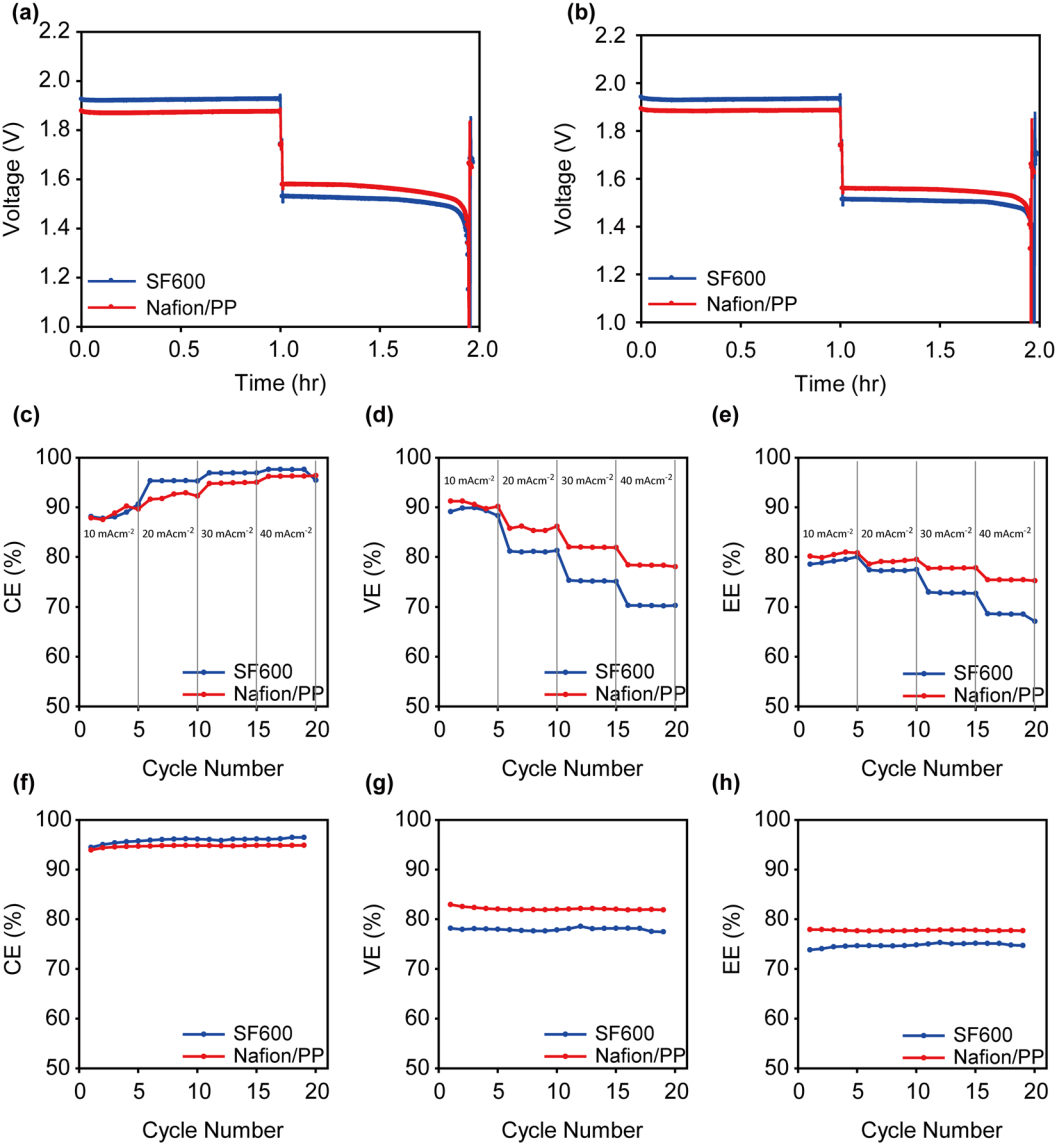
The charge discharge curves of the Nafion/PP and SF-600-based ZBB single cells at (**a**) the first cycle and (**b**) 19^th^ cycle. (**c**) Coulombic, (**d**) voltage, and (**e**) energy efficiencies of the Nafion/PP and SF-600-based ZBB single cells with various current densities from 10 to 40 mAcm^−2^. (**f**) Coulombic, (**g**) voltage, and (**h**) energy efficiencies of the Nafion/PP and SF-600-based ZBB single cells with cycling at 20 mAcm^−2^.

To demonstrate the practical applicability of the Nafion/PP membrane, ZBB single cell performances with the Nafion/PP membrane were measured and compared with those of the cell with the SF-600 membrane. The charge-discharge curves of the two single cells at the first and 19^th^ cycle are shown in Fig. 4a,b, respectively. At the first cycle, it is evident that the charging voltages were lower by 50 mV and discharging voltages higher by 50 mV for the Nafion/PP membrane, indicating smaller polarizations. Since identical electrodes were used for the two cells, the observed difference should attribute to the membrane. The polarization result is in good agreement with that from the ASR difference (Fig. 3c); the smaller ASR for the Nafion/PP membrane is dictated by the smaller polarizations. The discharge capacities were nearly identical, which is in accordance with the comparable Br_2_ crossover rate for the two membranes. At the 19^th^ cycle, the voltage profiles were nearly unchanged from those measured at the first cycle, which suggests a high stability of the ZBB cells designed in this work.

For the Nafion/PP and SF-600-based ZBB single cells, a rate capability test was conducted at various current densities (10, 20, 30, and 40 mAcm^−2^). Fig. 4c–e show the coulombic, voltage, and energy efficiencies of the two cells with various current densities. Since the charging capacity was fixed to 120 mAh, the degree of self-discharge was reduced with increasing current density due to the shortened charging time, which consequently increased coulombic efficiencies. On the contrary, in the case of the voltage efficiencies, the increase of overvoltage with current density led to the reduction in voltage efficiencies. The comparison of the Nafion/PP and SF-600 membranes confirms that the Nafion/PP membrane has superior rate capability; in all the current densities investigated, the voltage efficiencies of the Nafion/PP membrane were higher than those of the SF-600.

The coulombic, voltage, and energy efficiencies of the two cells during 19 cycles were displayed in Fig. 4f–h, respectively. For quantitative comparison, average efficiencies were calculated for the efficiencies from first to 19^th^ cycles. During the repeated cycling, the Nafion/PP membrane showed comparable coulombic efficiencies (94.7% for the Nafion/PP and 95.8% for the SF-600 membrane), which is in accordance with the similar Br_2_ flux between the two membranes (Fig. 3b). As expected from the considerable difference in ASR values, the voltage efficiency of the Nafion/PP membrane (77.7%) is higher by 4% than that of the SF-600 membrane (74.7%). As a combined effect of columbic and voltage efficiencies, the averaged value for energy efficiency was 82.1% for the Nafion/PP-based cell and 77.9% for the SF-600-based cell. It has been perceived in this field that dense ion exchange membranes are not suitable due to large voltage loss. However, this work verifies for the first time that the dense, ion exchange membrane can outperform the conventional porous membrane for the use as a ZBB membrane by significantly reducing the membrane thickness with inexpensive porous substrate.

**Figure 5:**
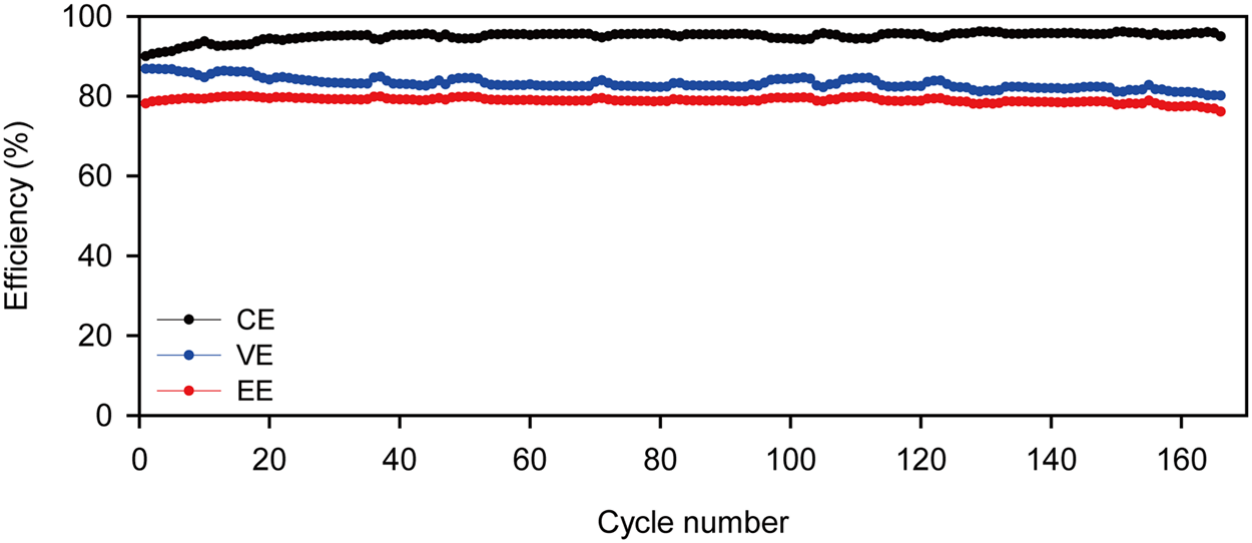
Coulombic efficiencies, voltage efficiencies, and energy efficiencies of the Nafion/PP-based ZBB single cell during an extended cycling at 20 mAcm^−2^.

In order to assess the long-term stability of the Nafion/PP membrane, the single cell with the membrane was operated for 166 cycles. As given in Fig. 5, the coulombic, voltage, and energy efficiencies were stably maintained over the 166 cycle operation. Since any membrane degradations lead to a change in coulombic and/or voltage efficiency for flow batteries, such stable operation indicates a high durability of the membrane during an extended operation.

It should be noted that the Nafion/PP membrane can be used for other redox flow batteries which requires both positive and negative ion transport and blocking of neutral redox material. The newly suggested flow batteries including TEMPO/Zn^17^ and organic redox couple-based aqueous flow batteries^18–20^ can employ the bi-ion conducting, non-porous composite membrane due to its low area specific resistance, high blocking function for redox materials, and high chemical and mechanical robustness.

## Conclusion

The use of ultra-thin, dense Nafion/PP membrane for ZBB was successfully demonstrated. The dense Nafion phase filled in the pores of PP separator enables the passage of Zn^2+^ and Br^−^ ion, but effectively blocks the crossover of Br_2_ through the membrane. The PP separator not only mechanically supports the thin membrane but also contributes to the cost reduction by considerably reducing the amount of Nafion used. The Nafion/PP membrane showed smaller ASR and analogous Br_2_ permeability in comparison with the commercial SF-600 porous membrane. As a result, the ZBB based on the Nafion/PP membrane exhibited higher voltage efficiency, comparable coulombic efficiency, and higher energy efficiency, demonstrating the efficacy of this approach in terms of performance.

## Experimental Methods

### Preparation of the pore-filled composite membranes

The Nafion/PP composite membrane was prepared with solution casting of a 10 wt% Nafion solution in N-Methyl-2-pyrrolidone (NMP) onto a 20 μm-thick porous PP substrate (SKC) with a porosity of 60%. The cast was dried in a 60 °C oven for 2 h. The membrane was equilibrated in water at 80 °C for 12 h before use to fully hydrate the impregnated Nafion ionomers.

### Solvation free energy calculation and dynamic light scattering

To design the Nafion solution, solvation free energy of Nafion in various solvents was calculated with fully atomistic model implemented using the Materials Studio (Biovia Inc.) software and COMPASS II commercial force field. We constructed 5 different Nafion + solvent models and each model has an identical Nafion chain and 300 of solvent molecules. Molecular dynamics simulations were proceeded using Forcite module in order to equilibrate the cell in terms of energy and density. The Ewald summation method with an accuracy of 10^−5^ kcal mol^−1^ was used to calculate the electrostatic interactions, and the atom based summation method was applied to determine the van der Waals interactions. The Berendsen algorithm and NHL algorithm was used to control the temperature and pressure of the cell, respectively. In order to equilibrate the model, the NPT molecular dynamics simulation was conducted for 200 ps with a temperature setup of 298 K. It was confirmed that multiple variables, such as energy and density, converge to specific values and are not changed much anymore. After the equilibrating process, solvation free energy was calculated during the NVT molecular dynamics for 200 ps.

Dynamic light scattering test were conducted to measure the dimension of Nafion in the casting solvents. The hydrodynamic radius (R_h_) of Nafion in each solvents were observed with Zetasizer nano ZS90 (Malvern Co.). All the samples were diluted into 1 wt% for precise measurement and were proceeded under identical condition of 25 °C and 90 °C of scattering angle.

### Membrane characterizations

The morphologies of the composite membranes were investigated by low kV scanning electron microscopy (Zeiss Merlin field-emission SEM) and the cross-section ion-milling method (GATAN Ilion II model 697). The area specific resistance (ASR) of the membranes was measured for a symmetric cell equilibrated with 0.5 vol.% Br_2_–containing ZBB electrolyte (2.25 M ZnBr_2_ + 0.5 M ZnCl_2_ + 0.8 M 1-Ethyl-1-methylpyrrolidinium bromide (MEP) + 5 ml L^−1^ Br_2_) by using electrochemical impedance spectroscopy over a frequency range of 1 Hz to 100 kHz (VSP potentiostat, Biologics). The value for ASR was determined from the increase in the Ohmic resistance by introducing a membrane in a conductivity cell. Br_2_ diffusivity in the membranes was measured using a diffusion cell having a solute reservoir (0.2 M Br_2_ in 2.25 M ZnBr_2_ + 0.5 M ZnCl_2_ solution) and a blank reservoir (2.25 M ZnBr_2_ + 0.5 M ZnCl_2_ solution). The concentration of the diffused Br_2_ in the blank reservoir was measured by a UV-vis spectrometer (Genesys 10 S, Thermo Scientific). From the time-dependent change in the Br_2_ concentration in the blank reservoir, Br_2_ molar flux and Br_2_ diffusivity were determined.

### ZBB single cell test

A ZBB single cell (active area: 2 × 3 cm^2^) consisted of a membrane, two carbon felt (Jiuhua Hi-Tech Co., Ltd) electrodes heat treated at 400 °C 3 h under ambient air, two bipolar plates, two copper current collectors, and PTFE gaskets. The electrolyte is composed of 2.25 M ZnBr_2_, 0.5 M ZnCl_2_, 0.8 M MEP, and 5 ml L^−1^ Br_2_. A single cell test was conducted with a battery cycler (WBCS3000, Won-A Tech). The electrolyte volume was 20 mL for both positive and negative electrolytes and the flow rate was 40 mL min^−1^. The cells were charged at 20 mAcm^−2^ with a capacity cut of 120 mAh and discharged at 20 mAcm ^−2^ with a cut-off voltage of 0.01 V. ZBBs usually operate at low current densities due to the relatively large polarization from the positive electrode and large internal resistance. Rate capability was assessed by changing current density from 10 to 40 mAcm ^−2^ at a fixed charge capacity of 120 mAh. At each current density, the cell was operated 5 cycles.

## Electronic supplementary material

Supporting Information
