# Supplementary material for: Ultrathin Nafion-filled porous membrane for zinc/bromine redox flow batteries
Source: Sci Rep. 2017 Sep 5;7:10503. doi: 10.1038/s41598-017-10850-9 (PMC5585230; doi:10.1038/s41598-017-10850-9)
Supplement: Supplementary file 1 — Supporting Information [file 41598_2017_10850_MOESM1_ESM.pdf]

Supporting information

of

Ultrathin Nafion-filled porous membrane for zinc/bromine redox flow batteries

Riyul Kim[1†], Hyun Gyu Kim[1†], Gisu Doo[1], Chanyong Choi[1], Soohyun Kim[1], Ju-Hyuk Lee[1], Jiyun Heo[1], Ho-Young Jung[3], and Hee-Tak Kim[1,2*]

[1]*Department of Chemical and Biomolecular Engineering, Korea Advanced Institute of Science and Technology (KAIST), 291, Daehak-ro, Yuseong-gu, Daejeon, 34141, Republic of Korea*

[2]*Advanced Battery Center, KAIST Institute for the NanoCentury, Korea Advanced Institute of Science and Technology (KAIST), 335 Gwahangno, Yuseong-gu, Daejeon 34141, Republic of Korea*

[3]*Department of Environment & Energy Engineering, Chonnam National University, 77, Yongbong-ro, Buk-gu, Gwangju, 61186, Republic of Korea*

*Corresponding author: heetak.kim@kaist.ac.kr

[†]The authors have contributed equally to this work.

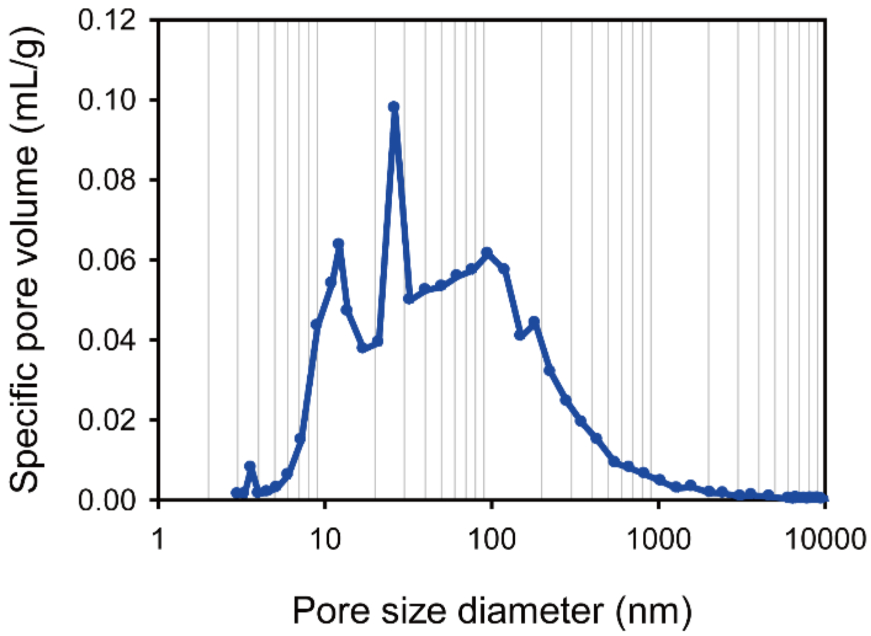

Figure S1. Pore size distribution of the SF600 membrane

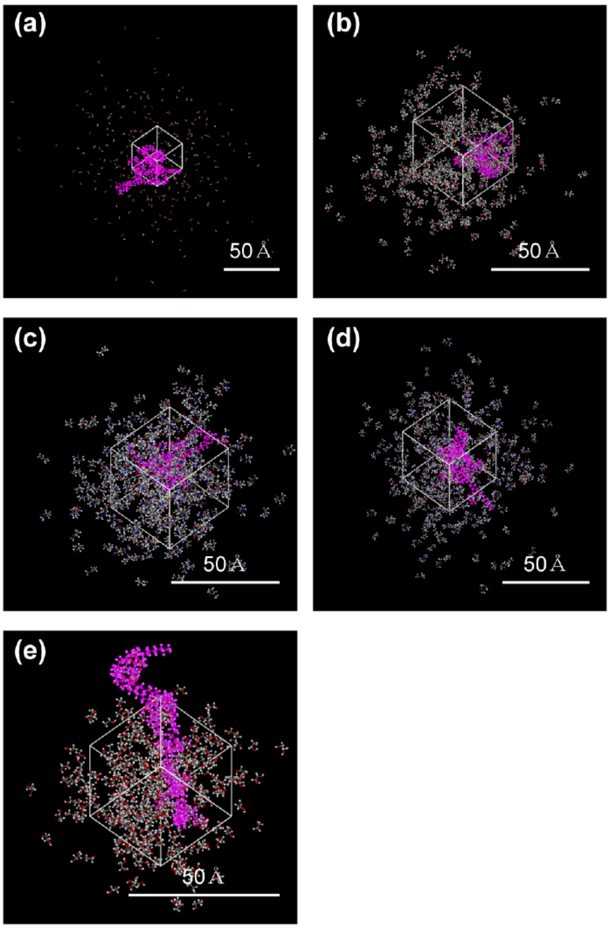

Figure S2. Snapshots of Nafion+solvent model; solvent molecules are (a) water, (b) IPA, (c) NMP, (d) DMAc, (e) ethylene glycol, respectively. (Purple: Nafion chain, others: solvent molecules)

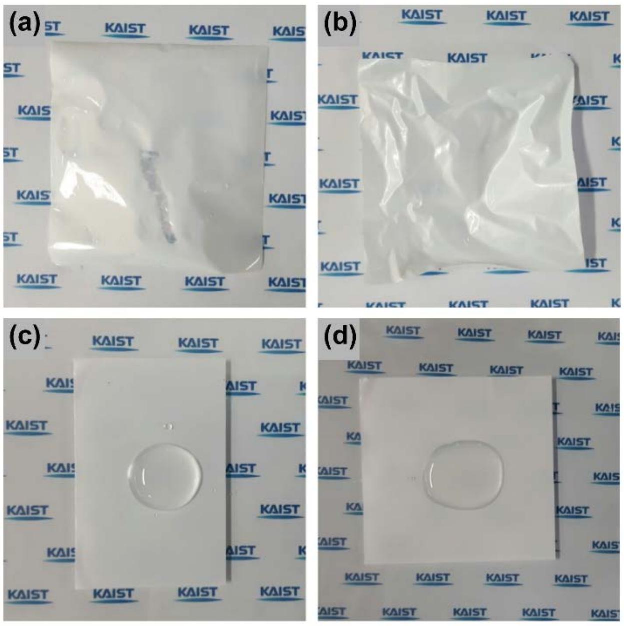

Figure S3. Optical image of the Nafion-filled PP membrane based on (a) DMAc (b) IPA (c) water (d) EG as a casting solvent

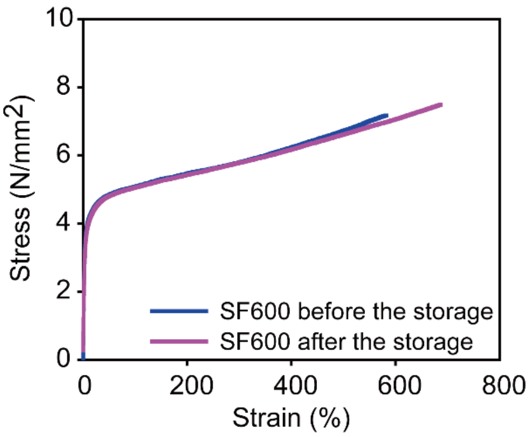

Figure S4. Stress-strain curves for the SF600 membrane before and after the Br_2 storage for 24 h
